# Piezoelectric and Magnetoelectric Thick Films for Fabricating Power Sources in Wireless Sensor Nodes

**DOI:** 10.3390/s90806362

**Published:** 2009-08-17

**Authors:** Shashank Priya, Jungho Ryu, Chee-Sung Park, Josiah Oliver, Jong-Jin Choi, Dong-Soo Park

**Affiliations:** 1 Center for Energy Harvesting Materials and Systems (CEHMS), Dept. of Materials Science and Engineering, Virginia Polytechnic Institute and State University, Blacksburg, VA 24061, USA; E-Mails: pcs94@vt.edu (C.-S.P.); joolive1@vt.edu (J.O.); 2 Functional Ceramics Research Group, Korea Institute of Materials Science (KIMS), Changwon, Gyeongnam 641-831, Korea; E-Mails: jhryu@kims.re.kr (J.R.); pds1590@kims.re.kr (D.-S.P.)

**Keywords:** piezoelectric, magnetoelectric, energy harvesting, thick films, MEMS

## Abstract

In this manuscript, we review the progress made in the synthesis of thick film-based piezoelectric and magnetoelectric structures for harvesting energy from mechanical vibrations and magnetic field. Piezoelectric compositions in the system Pb(Zr,Ti)O_3_–Pb(Zn_1/3_Nb_2/3_)O_3_ (PZNT) have shown promise for providing enhanced efficiency due to higher energy density and thus form the base of transducers designed for capturing the mechanical energy. Laminate structures of PZNT with magnetostrictive ferrite materials provide large magnitudes of magnetoelectric coupling and are being targeted to capture the stray magnetic field energy. We analyze the models used to predict the performance of the energy harvesters and present a full system description.

## 1. Introduction

Wireless sensor nodes are used in a wide spectrum of applications, ranging from the human body to the oceans to industrial machines. One can envision a personal health monitoring system where external sensor nodes measure the body temperature, pulse rate, and blood pressure and transmit the data to a PDA or another hand held wireless devices. Sensor nodes can also be implanted in the human body to monitor glucose levels and toxins, while communicating with outside control devices. In the ocean environment there are a variety of deployable wireless sensor networks that conduct operations such as surveillance, chemical and biological studies, and oil exploration. In case of industrial applications, a variety of sensors are utilized to monitor process and manufacturing operations including, gas, chemical, temperature, strain, humidity, motion, structural health, and explosives. In all these applications the lifetime of the sensor node is limited by the size of battery. In the case of implanted devices, the battery is inaccessible and thus other methods for powering the sensor nodes are required.

The advancement in CMOS-technology, IC manufacturing, and networking techniques utilizing Bluetooth communication have significantly reduced the total power requirements of wireless sensor nodes. The development of ultra-low-power components has lead to power requirements which are extremely small (microcontrollers ∼160 μA/MHz, sensor ∼120 μA, transceiver (RS-232) ∼3 mA, and transceiver (RS-485) ∼120 μA) [1]. Recently, results have been reported on the development of wireless sensor nodes requiring power consumption of a few hundred microwatts. Such nodes will form the future of dense *ad hoc*-networks transmitting data from 1 to 10 meters. For communication over 10 meters, the sensor networks are projected to operate in a multi-hop fashion, replacing large transmission distances with multiple low power–low cost nodes [2–8]. In order for the nodes to be conveniently placed and used they should be as small as possible, which puts an upper limit on their lifetime. If an electronic device with a 1 cm^3^ non-rechargeable lithium battery (at a max energy density of 2,880 J/cm^3^ or 800 watt hour per liter) were to consume on average 100 μW of power, the device would last 333 days. A lifetime of approximately one year is not practical [9]. Even though the nodes in the wireless network will be much smaller than the 1 cm^3^ area, the power requirements will force them to use a battery of much larger size, enhancing the system volume. Clearly, there is an acute need for the development of alternative power sources [10,11].

The power requirement for commercially available transceivers such as Crossbow’s Mica series, Sentilla nodes and Dust Network nodes, varies between 25–150 mW in the active state and less than 10 mW in the sleep state. This power is consumed by processors, radio, and sensors, depending upon factors such as transmission and receive rate. The power consumed by processors can range from 0.2 nJ/instruction to 2 nJ/instruction at 35 kHz to 400 MHz. The power consumed by radio lies in the range of 150 nJ/bit for short distances. The sensor power requirement depends upon the mechanism utilized such as magnetic, piezoelectric, capacitive, etc. Piezoelectric and magnetoelectric sensors have the advantage that they are passive and consume power only for processing and radio, thus further reducing the energy budget.

In general, there are four possible ways to address the problem of powering the wireless sensor nodes, as follows: (1) enhance the energy density of storage systems; (2) reduce the power consumption of wireless nodes; (3) develop self-powered nodes by generating or scavenging power and (4) develop other novel methods for powering the nodes. Out of these various possible solutions the most efficient and practical method is to develop self-powered nodes by scavenging energy from the wasted ambient energy. Table I shows the list of mechanical energy sources available in various scenarios which can be trapped for generating electricity locally. Recently, our focus has been on industrial machines as the source of energy and also the platform for implementing wireless health monitoring sensor network. In addition to vibrations, industrial machines are also source of stray magnetic fields which can be trapped for generating electricity using magnetoelectrics. Thus, the same device can convert both mechanical and magnetic energy into electricity.

**Figure 1.**
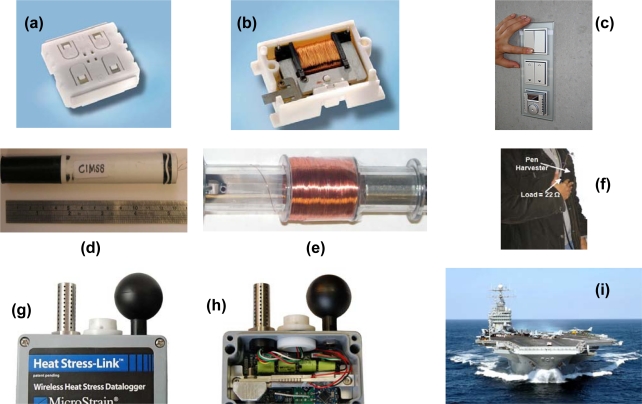

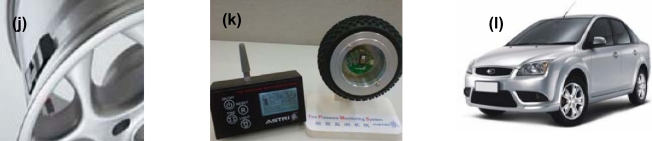
Demonstrated vibration energy harvesting systems. (a)–(c) enocean^®^ “Pushbutton Transmitter Module” (PTM 200), ECO 100 harvester, and wall mounted switch (Website: http://www.enocean.com/); (d)–(f) Virginia Tech’s “pen” and integrated pen harvester prototype generating continuous power of 0.46–0.66 mW under normal human actions; (g)–(i) vibration energy harvesting heat stress nodes developed by MicroStrain Inc. (Website: http://www.microstrain.com) for Navy applications (taken from: Energy Harvesting Technologies, Ed. S. Priya and D. Inman and http://www.maritimequest.com/); and (j)–(l) tire pressure monitoring system (TPMS) developed by ASTRI for automobiles (Website: http://www.astri.org).

Several commercial energy harvesting prototypes addressing the needs in industries ranging from housing to aircraft to industrial process monitoring systems have been demonstrated. Figure 1 shows some of the prototypes that have been deployed on various platforms. Figure 1(a) shows the picture of enocean^®^ “Pushbutton Transmitter Module” (PTM 200) which has been implemented in wall-mounted electrical switches as shown in Figure 1(c). The transmitter generates power using the energy harvester similar to one shown in Figure 1(b) (ECO 100) which converts linear motion into electricity using an electromagnetic induction mechanism. The dimensions of harvester are 33.3 × 22.0 × 10.8 mm^3^ and it can provide output pulse of up to 5 V from a force of 5 N with travel distance of 2 mm. Figure 1(c) shows the picture of a wall switch where devices like ECO 100 can find application. Figure 1(d) shows the picture of Virginia Tech’s “pen” which was found to generate power of 3 mW at 5 Hz and 1 mW at 3.5 Hz operating under displacement amplitude of 16 mm (corresponding to an acceleration of approximately 1.14 g_rms_ at 5 Hz and 0.56 g_rms_ at 3.5 Hz respectively) [12]. The pen utilizes Faraday’s law of electromagnetic induction where a magnet oscillates inside a wound coil as shown in Figure 1(e). The integrated pen harvester prototype was found to generate continuous power of 0.46–0.66 mW under normal human actions such as jogging and jumping, as shown in Figure 1(f). Figure 1(g),(h) shows photographs of vibration energy harvesting heat stress nodes developed by MicroStrain Inc. for Navy applications. The module consists of miniature relative humidity (RH) sensor, a dry bulb temperature sensor and black body temperature sensor that combined provide data for determining each ship compartments’ wet bulb globe temperature (WBGT) and heat stress indexes. The vibration harvester was attached to an air compressor and tuned to work at 52 Hz sinusoidal vibration of amplitudes 30–40 milliG’s which was the predominant vibration frequency of machine. Figure 1(i) shows the picture of ship just to give an idea of working platform in terms of dimensions. Figure 1(j–k) shows picture of a tire pressure monitoring system (TPMS) developed by ASTRI [13,14]. Figure 1(l) shows the TPMS application platform where tire vibrations are being used to monitor pressure and transmit the data wirelessly. All these results shown in Figure 1 clearly demonstrate the promise of vibration energy harvesting technologies.

## 2. Vibration Energy Harvesting

**Figure 2.**
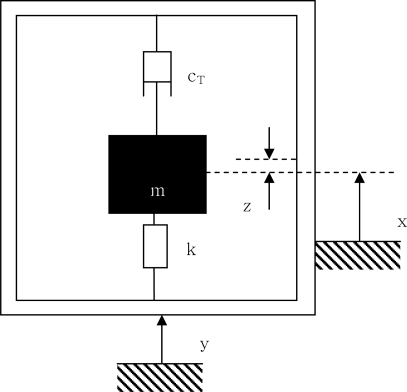
Diagram of a spring-mass-damper base excitation system.

Traditionally, a single degree of freedom (SDOF) vibrating mass-spring-damper base excitation system has been used to describe the magnitude of energy that can be harvested from a vibration source as shown in Figure 2. The equation of motion for the vibrating system is given as:

(1) $$m\ddot{x}(t)+c_{T}\;[\dot{x}(t)-\dot{y}(t)]+k[x(t)-y(t)]=0$$

where stiffness of spring is *k*, total amount of damping (electrical and parasitic mechanical) is *c_T_*, seismic mass is *m,* displacement of the base is given by *y*, and the displacement of seismic mass is given by *x*.

Equation (1) can be rearranged in order to derive a differential equation for the relative motion, *z*(*t*), as a function of the base acceleration:

(2) $$m\ddot{z}(t)+c_{T}\;\dot{z}(t)+kz(t)=-m\ddot{y}(t)$$

where *z*(*t*) is the relative motion of seismic mass with respect to housing. Using Equation (2), it can be shown that the total power dissipated in damper under sinusoidal forcing is given as:

(3) $$P_{T}\;(\omega )=\frac{m\zeta_{T}\;Y^{2}{\left(\frac{\omega }{\omega_{n}}\right)}^{3}\;\omega^{3}}{{\left[1-{\left(\frac{\omega }{\omega_{n}}\right)}^{2}\right]}^{2}+{\left[2\zeta_{T}\;\left(\frac{\omega }{\omega_{n}}\right)\right]}^{2}}$$

where *ξ_T_* is the total damping ratio of the system. At resonance, the total power in the system can be split into sum of mechanical power dissipated and the electrical power generated:

(4) $$P_{T}\;(\omega )=P_{m}\;(\omega )+P_{e}\;(\omega )=\frac{m\zeta_{m}\;Y^{2}\;\omega_{n}{}^{3}}{4{\left(\zeta_{m}+\zeta_{e}\right)}^{2}}+\frac{m\zeta_{e}\;Y^{2}\;\omega_{n}{}^{3}}{4{\left(\zeta_{m}+\zeta_{e}\right)}^{2}}$$

where the electrical power generated is equal to *P_e_(ω)*, the mechanical power dissipated is given by *P_m_(ω)*, and *ξ_e_* and *ξ_m_* are the electrical and mechanical damping ratios respectively of the harvester. The maximum power which can be generated by the electrical power takeoff system occurs when the electrical damping is equal to mechanical damping (*ξ_e_* = *ξ_m_*). Therefore the maximum electrical power which can be generated is given as [10]:

(5) $$P_{e,\text{max}}\;\left(\omega_{n}\right)=\frac{mY^{2}\;\omega_{n}{}^{3}}{16\zeta_{m}}$$

Equation (5) represents the theoretical maximum amount of electrical power which can be dissipated in electrical load. Depending upon the amplitude, frequency range, operating temperature range, and lifetime, any of the five mechanisms, namely electromagnetic, piezoelectric, electrostatic, magnetoelectric and electrets, can be selected to convert available vibration energy into electricity. In this manuscript, we review the developments made in the field of piezoelectric and magnetoelectric energy harvesting, mainly focusing on thick films.

**Figure 3.**
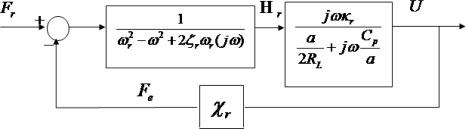
Block diagram for response around r^th^ mode of the parallel (a = 1) and series (a = 2) connected piezoelectric bimorph.

Materials performance plays key role in the design of harvester. Detailed analytical model for piezoelectric energy harvesting using bimorph transducer has been proposed by Oliver and Priya. Figure 3 shows the block diagram form of a solution to illustrate the effect of mechanical system on electrical output and the feedback term with which the electrical system affects the mechanical vibration of system. The variables *H_r_*(*s*), *F_r_*(*s*) and *U*(*s*) are Laplace transforms of modal forcing, modal displacement, and output voltage. Using the block diagram, a transfer function from input forcing function to output voltage and displacement can be calculated. The transfer function from input base excitation force to output current can be calculated as:

(6) $$U(t)=\frac{G}{1+\mathrm{GH}}F_{r}\;(t)$$

where G is the through path which represents the generation of current from mechanical motion, and H is the feedback path which represents the electrical damping that system places on structure.

The output voltage and power for a series (a = 2) and parallel (a = 1) bimorph around the *r^th^* mode can be reduced to the following equations [15]:

(7) $$U(t)=\left(\frac{j\omega \kappa_{r}\;\left(\frac{M_{b}}{L}\;\int_{0}^{L}\Phi_{r}\;(x)\mathrm{dx}+M_{t}\;\Phi_{r}\;(L)\right)}{(\frac{a}{2R_{L}}+\frac{C_{p}}{a})(\omega_{r}^{2}-\omega^{2}+j2\zeta_{r}\omega_{r}\omega )+j\omega \kappa_{r}\;\chi_{r}}\right)\left(\omega^{2}\;Y_{0}\;e^{j\omega t}\right)$$

$$P_{\mathrm{avg}}\;(t)=\frac{U^{2}}{2R_{L}}$$

where Φ*_r_*(*x*) is the mode shape of the r^th^ mode of cantilever beam, *L* is the length of beam, *M_t_* is the seismic mass, *M_b_* is mass of beam, *U* is the voltage across the load resistance and P_avg_ is the power. The modal mechanical forcing term is given as:

(8) $$f_{r}\;(t)=-\frac{M_{b}}{L}\;\frac{d^{2}\;y(t)}{{\mathrm{dt}}^{2}}\;\int_{0}^{L}\;\Phi_{r}\;(x)\mathrm{dx}-M_{t}\;\Phi_{r}\;(L)\frac{d^{2}\;y(t)}{{\mathrm{dt}}^{2}}$$

The modal coupling term transducing modal velocity to current in the electrical equation is given as [15]:

(9) $$\kappa_{r}=\frac{d_{31}\;h_{\mathrm{pc}}\;b}{s_{11}^{E}}\;\int_{0}^{L}\;\frac{d^{2}\;\Phi_{r}\;(x)}{{\mathrm{dx}}^{2}}\;\mathrm{dx}=\frac{d_{31}\;h_{\mathrm{pc}}\;b}{s_{11}^{E}}\;\frac{d\Phi_{r}\;(x)}{\mathrm{dx}}|{}_{x=L}$$

where *h_pc_* is the distance from the ceramic centerline to the neutral axis. The backward modal coupling creating the electrical damping on mechanical structure is given as:

(10) $$\chi_{r}=\vartheta \;\frac{d\Phi_{r}\;(x)}{\mathrm{dx}}\;{|}_{x=L}=\frac{1}{s_{11}^{E}}\;\frac{d_{31}\;b}{{\mathrm{ah}}_{p}}\;\left(\frac{h_{s}^{2}}{4}-{\left(h_{p}+\frac{h_{s}}{2}\right)}^{2}\right)\;\frac{d\Phi_{r}\;(x)}{\mathrm{dx}}{|}_{x=L}$$

where *h_s_* and *h_p_* are the thicknesses of the piezoceramic and substructure layers, $s_{11}^{E}$ is the modulus of elasticity for the ceramic, *d*_31_ is the piezoelectric constant, b is the width of the beam, and a = 1 (for bimorph layers connected in parallel), a = 2 (for bimorph layers connected in series) [15]. The capacitance of bimorph is given as:

(11) $$C_{p}=\frac{\varepsilon \mathrm{bL}}{h_{p}}$$

Using Equations (7)–(11), it can be shown that piezoelectric material with high *d*_31_, and *g*_31_, and low loss is required for bimorph transducer. The loss in piezoelectric material is mainly related to dielectric and electromechanical losses. These parameters contribute to the electrical damping.

**Figure 4.**
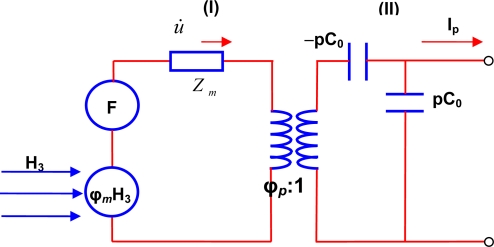
Equivalent circuit model for magnetoelectric energy harvesting.

Dong *et al*. have presented the equivalent circuit model for magnetoelectric energy harvester as shown in Figure 4. Based upon this model, the induced voltage (*V*_induced_) across the dielectric layer under open circuit condition can be given as [16]:

(12) $$V_{\mathrm{induced}}=-\phi_{p}\;\left(\frac{Z_{c}}{Z_{m}}\right)\left(F+\phi_{m}\;H\right)$$

where *φ*_p_ is the electromechanical coupling factor, *φ*_m_ is the magneto-elastic coupling factor, Z_C_ is the capacitance impedance (Z_c_ = 1/*jωpC*_0_) and *Z*_m_ is the mechanical impedance. The negative “—” sign indicates the reversal of phase between the applied F (or H) and the induced voltage V_induced_. It can be seen from Equation (12) that a high electromagnetic coupling and magneto-elastic coupling is required to harvest the vibration and magnetic field simultaneously.

## 3. Piezoelectric Thick Films for Energy Harvesting

**Figure 5.**
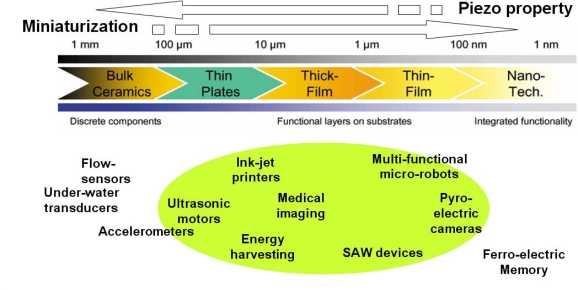
Application areas of piezoelectric materials with varying thickness.

Figure 5 highlights the application spectrum of piezoelectric thin/thick films. Piezoelectric thick films with thickness range of 1–100 μm have been used in devices such as micro-fluidics, micropumps, accelerometers, and energy harvesters. In addition to energy harvesters, other microelectromechanical systems (MEMS) such as accelerometers, acoustic sensors, and infrared detectors also require dense, crack-free piezoelectric thick films [17–25]. However, synthesis of thick films is complex, as it is more susceptible to cracks by thermal stresses induced by difference in thermal expansion coefficients between the film and substrate [26,27]. Another important parameter for enhancement of ferroelectric and piezoelectric properties of films is texture. It is well known that electromechanical properties of Pb(Zr,Ti)O_3_ (PZT) film strongly depends on crystallographic orientation [28–30]. Piezoelectric properties of (001) oriented rhombohedral PZT films near morphotropic phase boundary (MPB) are superior than those of (111) oriented films over the entire composition range [31–35].

In order to fabricate high quality thin/thick PZT films, chemical or physical deposition methods such as sputtering, pulsed laser deposition (PLD), metal organic chemical deposition (MOCVD), sol-gel, tape casting, and screen printing have been employed [36–39]. Published techniques for synthesis of PZT thin films can be divided into two categories, those that use *in-situ* crystallization (i.e., crystallization during deposition) and those that involve post-deposition crystallization. MOCVD and physical deposition at elevated temperatures fall into the former category [36]. For *in-situ* crystallization, oxygen partial pressure is known to be a critical process control parameter. The second category includes most chemical and low temperature physical deposition techniques [37–39]. Pre- and post-crystallization processes are also known to influence the nucleation, microstructure, texture, and electrical properties of PZT films.

**Figure 6.**
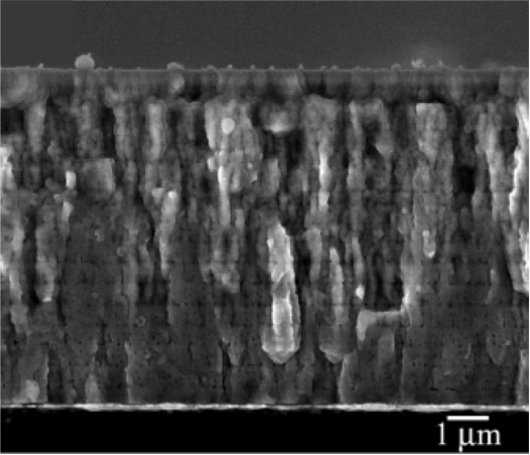
Cross-sectional image of 8 μm-thick (100) oriented PZT film using sol-gel [41].

Synthesis of crack-free thick films by using PLD, sputtering, and sol-gel requires careful optimization of various synthesis parameters [40–43]. Park *et al*. have reported (100)-oriented 8 μm crack-free thick film using sol-gel route, as shown in Figure 6 [41]. By controlling the pyrolysis steps, they were able to obtain preferred orientation and by using an organic additive [polyvinylpyrrolidone (PVP)] they were able to increase the thickness of film. Using the combination of these two parameters, 8-μm-thick films with (111) or (100) texture were successfully synthesized.

**Figure 7.**
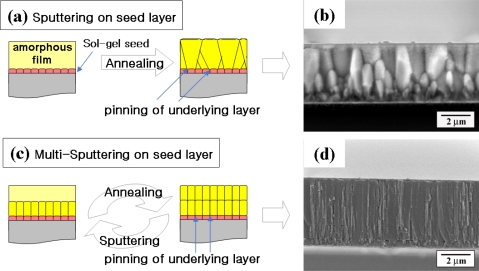
Sputtering method with seed layer to control the grain growth. (a) schematic diagrams of normal sputtering on a seed layer, (b) cross-sectional view of 3.5 μm-thick film using normal sputtering on a seed layer [42], (c) schematic of multi-sputtering on a seed layer, and (d) cross-sectional view of 5 μm-thick film using multi-sputtering on a seed layer [43].

A combinatory process of sputtering and sol-gel with controlled nucleation and growth has been used to synthesize 5 μm thick (100)-oriented high quality films exhibiting longitudinal piezoelectric coefficient of >300 pC/N [42,43]. The microstructure and orientation of films was adjusted by first synthesizing a thin seed layer derived from sol-gel method, and then subsequent depositions by sputtering. The seed layer had the same composition as other PZT layers. Initially, when a sputtered thin layer was deposited on seed layer, the film had small grains with columnar structure; however, as the deposited film became thicker, it developed a large non-columnar grain structure with lateral growth, as shown in Figure 7a,b. The size of grains near the surface was larger than that near the substrate, indicating that grain boundary pinning effect becomes smaller as the distance from seed layer increases. Based on this observation, a multi-sputtering process was developed [43].

By reducing the thickness of film in each deposition step, its columnar microstructure was maintained, as shown in Figure 7c,d. Simultaneous optimization of both seed layer and multi-step sputtering process allowed suppressing the crack generation process. It was found that when the film had small in-plane grain size and fibrous columnar structure, the crack generation process was suppressed due to increase in strength and structural stability. Consequently, thickness of PZT film with (100) orientation was markedly increased up to 5 μm which also increased the piezoelectric properties [43].

**Figure 8.**
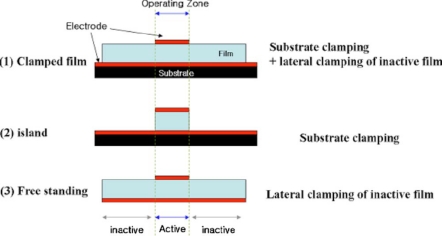
Schematic diagram illustrating two different types of clamping in fully-clamped, island, and freestanding films [48].

Piezoelectric properties were found to improve as the film thickness was increased [41–43]. This phenomenon is mainly related to reduction in clamping and damping effects of substrate [44–47]. Substrate clamping restricts the domain motion under applied electric field. However, it is difficult to directly associate the change in piezoelectric properties with degree of clamping in films, because these properties also depend of other microstructural variables such as grain size, grain shape, porosity, and texture [41,44–47]. The degree of clamping is often approximated by measuring the residual stress in films [41,47]. Substrate clamping can be reduced if the films are in free-standing state [48]. Recently, we have demonstrated systematic change in piezoelectric properties by synthesizing and measuring properties of three separate structures, clamped, island, and free-standing, as shown in Figure 8 [48]. The results showed that both lateral clamping as well as substrate clamping play an important role in controlling the ferroelectric response.

**Figure 9.**
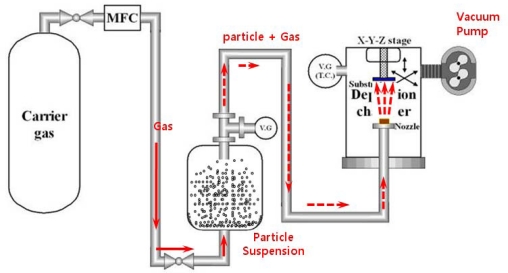
Schematic diagram of an aerosol deposition (AD) system.

Akedo *et al*. have introduced aerosol-deposition (AD) technique for synthesizing thick films [49,50]. This technique can provide crack-free dense thin and thick films with thicknesses ranging from submicrometer to several hundred micrometer with very fast deposition rates. Figure 9 shows a schematic diagram of an AD system, which consists of a carrier gas supply system with mass flow control, powder chamber containing the ceramic powder, and deposition chamber with motored X–Y stage and nozzle evacuated by rotary vacuum pump with mechanical booster. These three parts are all connected by a tube. The aerosol chamber contains the starting powders, which are mixed with a carrier gas to form an aerosol. The deposition chamber is devised for film formation. This chamber is connected with a vacuum system including a rotary vacuum pump and a mechanical booster pump. During deposition, the deposition chamber is evacuated by the vacuum system, and therefore a pressure difference between the aerosol chamber and deposition chamber is produced. The aerosolized ceramic particles from the aerosol chamber are delivered to the deposition chamber by carrier gas due to the pressure differential between the two chambers. The particles are accelerated and ejected through a slit-type nozzle, impacted onto a substrate to form a dense film in the deposition chamber. Particle velocity can be controlled by the carrier gas flow rate. The desired film thickness and deposition area can be obtained by scanning the substrate on motorized X-Y-Z stage. Fine patterning for the film is also possible by inserting a mask between the nozzle and the substrate.

AD is called as room temperature impact consolidation (RTIC) because dense films are formed by collision of fine particles with substrate at room temperature. However, actual deposition mechanism has not yet been established [49,50]. Considering previous studies on AD, it can be simply presumed that during collision with substrate, particles are broken into smaller pieces, rebound and impact each other, and then form the continuous film [49,50]. Consequently, the deposition of particles by AD appears to be largely dependent on kinetic energy and fracture energy of the primary particle, and therefore particle diameter and mechanical properties of particle such as strength are considered as important factors in producing dense high-quality AD film [49].

**Figure 10.**
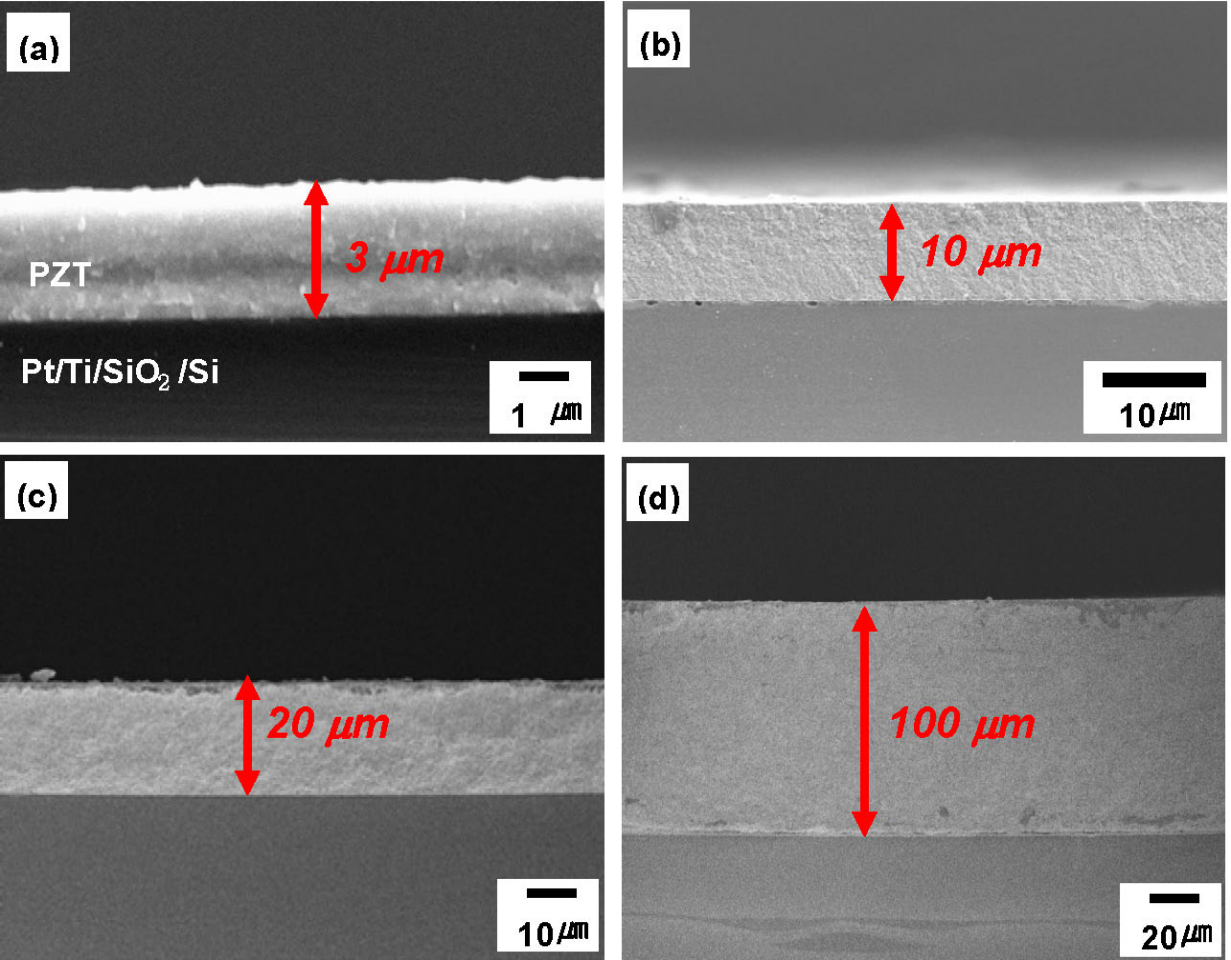
Cross-sectional view of piezoelectric film with various thickness by AD; (a) 3 μm, (b) 10 μm, (c) 20 μm, and (d) 100 μm [54].

**Figure 11.**
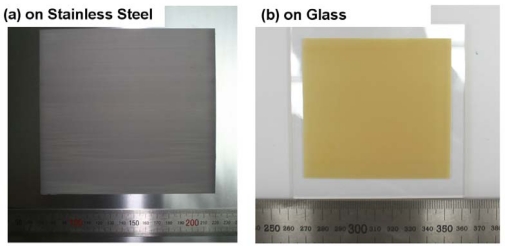
Large area piezoelectric 10 μm-thick films by AD. (a) 150 × 150 mm^2^ on stainless steel and (b) on 100 × 100 mm^2^ glass.

Park *et al*. have implemented this technique on various material systems including PZT-based compositions and realized highly dense and well-crystallized piezoelectric thick films of up to 100 μm thickness as shown in Figure 10 [51–54]. In terms of deposition area, PZT films up to 150 × 150 mm^2^ can be fabricated by AD with a 150 mm nozzle, as shown in Figure 11. Recently, 300 × 300 mm^2^ TiO_2_ photocatalytic thin films have been deposited by AD, which indicate the future possibility of fabricating PZT films on 12” wafers. AD-piezoelectric thick films exhibit excellent piezoelectric properties due to high density and minimized substrate clamping. AD process can also be used to induce dopants into the film in order to tailor the specific properties. For example, Zhang *et al*. [55] have reported that Mn doping in PZT thin films increases hysteretic properties and reduces fatigue. Recently, we have demonstrated hard piezoelectric (PZT-PZN-Mn) thick films (∼10 μm) by AD technique [56] which opens possibilities to design high energy density compositions.

### 3.1. Piezoelectric Micro-Generators for Wireless Sensor Nodes

Piezoelectric micro-generators have higher energy density compared to other mechanisms such as electrostatic, and electromagnetic ones [57–59]. PZT films have been widely used for fabricating harvesters because of their superior effective piezoelectric constant [24,25,60–63]. Jeon *et al*. demonstrated a d_33_ mode power generating device with interdigitated electrodes that can deliver 1.0 μW from 10.8g vibration amplitude at resonant frequency of 13.9 kHz [24]. However in most cases, the vibrations available for harvesting energy lie in the low frequency range of 50–150 Hz [58,59]. Fang *et al*. [60] have demonstrated a MEMS-based PZT cantilever structure with Ni proof mass that was found to generate 2.16 μW power from 1g vibration at the resonance frequency of 609 Hz. Shen *et al*. [25] have studied a d_31_ mode harvester with embedded Si proof mass which was found to generate 2.15 μW power from 2g vibration at resonance frequency of 461.15 Hz. Liu *et al*. [61] have reported micro-generators using varying length of beams in order to realize broadband behavior. The prototype was found to generate 3.98 μW power from 0.5 g vibration in the frequency range of 226 to 234 Hz. Renaud *et al*. [62] have reported a PZT based MEMS harvester with maximum power of 40 μW at 1.8 kHz. Important characteristics of reported MEMS based piezoelectric harvesters are summarized in Table 2.

**Figure 12.**
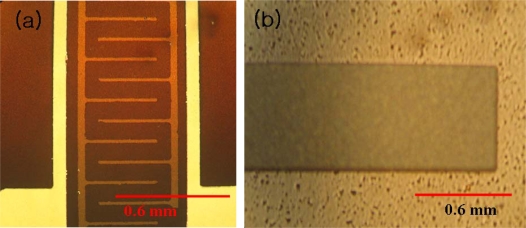
Plane Views of cantilevers: (a) d_33_ type and (b)d_31_ type.

**Figure 13.**
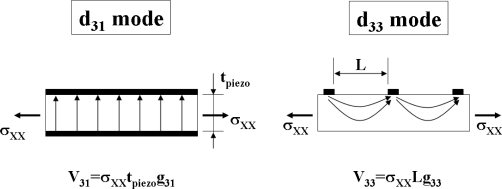
Two modes of piezoelectric conversion from input mechanical stress.

There are two piezoelectric modes (d_31_ and d_33_) commonly used for MEMS based piezoelectric transducers as shown in Figure 12(a),(b). Figure 13 shows the cross-sectional views of these piezoelectric modes [24,64] and Equations (13) and (14) are the representative relationship between stress σ_XX_ (or strain *x*_3_) and electric field E_i_ (or voltage V_3i_).

(13) $$x_{3}=d_{3i}\;E_{i}$$

(14) $$V_{3i}=\sigma_{\mathrm{xx}}\;g_{3i}\;L_{i}$$

where *x*_3_ is strain, *V*_3i_ is the open circuit voltage, *d*_3i_ (V/m) and *g*_3i_ (Vm/N) are piezoelectric constants, and *L_i_* is the distance between electrodes which could be either thickness of piezoelectric (*t*_piezo_) or *L*. The generated open-circuit voltage of a *d*_33_ type device will be much higher than that of the *d*_31_ type generator of similar beam dimensions.

Since the mass of a MEMS scale device is small, the operating frequency ranges are quite high, in the range of ∼kHz. In order to overcome this problem, the natural tendency has been to increase the tip mass of the cantilever [25,60,61]. However, this affects the mechanical integrity and lifetime of harvester. Choi *et al*. [65] have recently proposed various possible designs such as spiral to reduce the operating frequency range. This is an important area of research in MEMS harvester in order to expand the applicability in common scenarios.

## 4. Magnetoelectric Composites: Thick and Thin Film

Magnetoelectric (ME) effect can be described as an induced electric polarization in a material when a magnetic field is applied to it, or an induced magnetization in a material when an electric field is applied to it [66–69]. ME effect can be described as the product property of piezoelectric and piezomagnetic effects [70], or as the product property of pyroelectric and pyromagnetic effects [71]. Most of the ferromagnetic materials show magnetostrictive effect, however, piezomagnetic effect in these materials has not been observed. This implies that the strain caused by magnetic field in these materials is not linearly proportional to the field strength but to the square of magnetic field strength.

For energy harvesting applications, ME composite structures can be used to enhance the generated power from the micro-generator. The ME product property can be exploited to generate electricity from unused magnetic fields around electric motors and additionally from their mechanical vibrations. ME composites can also be used to convert the vibration energy into electricity with higher efficiency. In a simple design, the vibrations can be used to rotate a mechanical assembly which consists of magnets thereby creating the oscillating magnetic field [72]. Ferro Solutions has demonstrated a device based on this approach which was able to provide an energy density of 2.0 mW/cm^3^ at 21 Hz and 100 mG. There has been significant advances in improving the magnitude of ME coefficient of laminates which makes this technique promising. A combined magnetic and vibration energy harvesting device may be implemented on silicon using the thin film deposition methods and fabrication process flow described earlier and combining with micro-machining technique.

Recently, multiferroic nanocomposite thin films of ferroelectric and magnetostrictive materials have been reported motivated by the work of Zheng *et al.* [73–75], via physical deposition methods such as pulsed laser deposition (PLD) and chemical solution methods such as sol-gel spin coating. From microstructural point of view there are three kinds of nanostructured composite films, i.e., (i) 3-0 structures with magnetic spinel nanoparticles embedded in the ferroelectric films [76,77], (ii) 1–3 heterostructures (vertical heterostructures) consisting of magnetic spinel pillars vertically embedded into ferroelectric films [73,75], and (iii) 2–2 heterostructures (horizontal nanostructures) consisting of alternating layers of ferroelectric perovskite and magnetic oxide [78,79], as shown in Figure 12. Wan *et al.* [76] synthesized PZT-CoFe_2_O_4_ (CFO) composite thin film using sol-gel process and spin-coating technique. The films exhibited both good magnetic and ferroelectric properties, and the ME effect of these films was found to be strongly dependent on magnetic bias and magnetic field frequency. Zheng *et al.* [73–75] reported composite films where arrays of magnetic CFO nanopillars with diameters of 20–30 nm were embedded in a ferroelectric BTO matrix. Other combinations of PbTiO3–CoFe_2_O_4_ and BiFeO_3_–CoFe_2_O_4_ have also been grown on SrTiO_3_ single crystal substrates. These composite films have been found to exhibit excellent ferroelectric and ferromagnetic properties but there is no ME coupling. It seems that these structures have significant leakage which restricts the poling process. On the other hand, 3-0 and 2–2 heterostructures have been found to exhibit finite ME coupling due to the fact that leakage problem of magnetic phase can be avoided by controlling the volume fraction.

**Figure 15.**
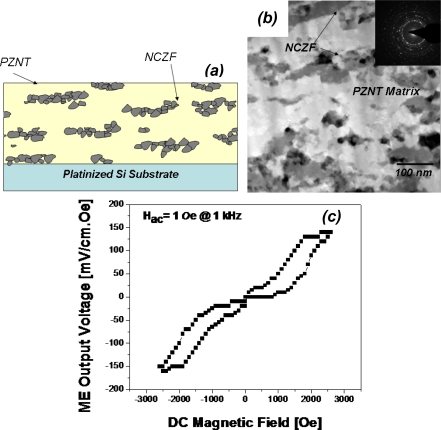
(a) schematics and (b) TEM microstructure of PZNT-NCZF 3-2 ME composite film by AD. (c) ME coefficient of 3-2 ME composite film with PZNT-NCZF fabricated by AD as a function of DC magnetic bias field [80].

Recently, we have successfully implemented AD process to fabricate ME composite thick films [80]. A highly dense 3-2 nanocomposite ME thick films of PZT-PZN and (Ni,Cu,Zn)Fe_2_O_4_ (NCZF) with thickness of over 10 μm was synthesized on platinized silicon substrate at RT. The schematic and TEM microstructure is shown in Figures 15(a–b). The fabricated nanocomposite film showed well dispersed laminated magnetic NCZF platelets inside PZT-PZN piezoelectric matrix. This structure eliminated the leakage problems found in 1–3 ME composite films and minimized substrate clamping effect, thus resulting in improved ME coefficient of 150 mV/cm·Oe as shown in Figure 15(c). In addition to ME characteristics, the deposition rate of ME films was exceptionally higher (over 1 μm/min) than other conventional thin film process.

## 5. Conclusions

We presented the review on prototype commercial vibration energy harvesters and their suitability for wireless sensor networks. A brief discussion was presented on modeling of vibration energy harvesters. Using the analytical models, it was shown that piezoelectric material with high d_31_ and g_31_, with low loss is required for bimorph transducer while high electromagnetic coupling and magneto-elastic coupling is required to harvest the vibration and magnetic field simultaneously using magnetoelectric composites. An in-depth discussion was provided on synthesis of thick films using AD. This is an extremely important development as large area deposition capability with excellent film quality will allow transitioning the micro-scale prototype devices. Combining the developments in the area of piezoelectric thick films with micro-machining techniques will allow fabrication of cost-effective energy harvesters.

## Figures and Tables

**Figure 14.**
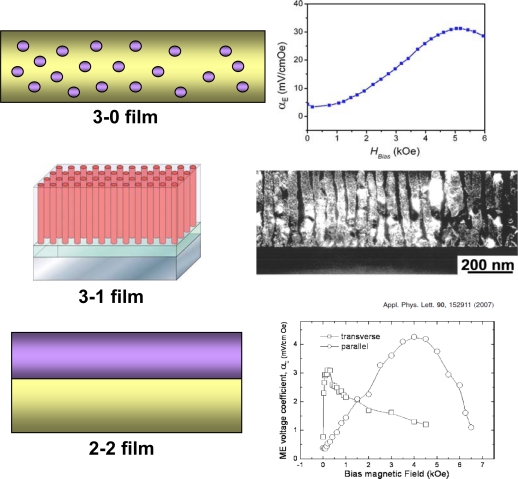
Reported ME composite film structures: 3-1 [73,74], 3-0 [76], and 2–2 [79].

**Table 1.** Sources of energy available in the surrounding which are/can be tapped for generating electricity [identified in first draft of standards on vibration energy harvesting, Center for Energy Harvesting Materials and Systems (CEHMS)].

**Human body**	**Vehicles**	**Structures**	**Industrial**	**Environment**
Breathing, blood pressure, exhalation, body heat, walking, arm motion, finger motion, jogging, swimming, eating, talking	Aircraft, UAV, helicopter, automobiles, trains, tires, tracks, peddles, brakes, shock absorbers, turbines	Bridges, roads, tunnels, farm house structures, control-switch, HVAC systems, ducts, cleaners, etc.	Motors, compressors, chillers, pumps, fans, conveyors, cutting and dicing, vibrating mach.	Wind, ocean currents, acoustic waves.

**Table 2.** Characteristics of reported piezoelectric micro-generators

Power (μW)	Frequency (Hz)	Acceleration (g)	Power density (μW/cm^3^)	Mode	Materials	Ref.
1.0	13.9k	10.8	37,037*	*d*_33_	PZT	[24]
2.16	609	1	10,843*	*d*_31_	PZT	[60]
2.15	461	2	3,272	*d*_31_	PZT	[25]
3.98	226–234	0.5	------	*d*_31_	PZT	[61]
40	1.8k	1.9*	21,680*	*d*_31_	PZT	[62]
0.045	1,495	2	-------	*d*_31_	AIN	[63]

* Estimated values
